# Optimizing the Efficiency of a Cytocompatible Carbon-Dots-Based FRET Platform and Its Application as a Riboflavin Sensor in Beverages

**DOI:** 10.3390/nano11081981

**Published:** 2021-07-31

**Authors:** Roberto Sotolongo-García, Eustolia Rodríguez-Velázquez, Manuel Alatorre-Meda, Mercedes T. Oropeza-Guzmán, Antonio Tirado-Guízar, Georgina Pina-Luis

**Affiliations:** 1 Tecnológico Nacional de México/Instituto Tecnológico de Tijuana, Centro de Graduados e Investigación en Química, Blvd. Alberto Limón Padilla S/N, Tijuana 22500, BC, Mexico; robertosg2989@gmail.com (R.S.-G.); oropeza@tectijuana.mx (M.T.O.-G.); antonio.tirado@tectijuana.edu.mx (A.T.-G.); 2 Facultad de Odontología, Universidad Autónoma de Baja California, Calzada Universidad 14418, Parque Industrial Internacional, Tijuana 22390, BC, Mexico; eustolia.rodriguez@uabc.edu.mx; 3 Tecnológico Nacional de México/I. T. Tijuana, Centro de Graduados e Investigación en Química-Grupo de Biomateriales y Nanomedicina, Blvd. Alberto Limón Padilla S/N, Tijuana 22510, BC, Mexico; 4 Cátedras CONACyT-Tecnológico Nacional de México/I. T. Tijuana, Centro de Graduados e Investigación en Química-Grupo de Biomateriales y Nanomedicina, Blvd. Alberto Limón Padilla S/N, Tijuana 22510, BC, Mexico; manuel.alatorre@tectijuana.edu.mx

**Keywords:** carbon dots, FRET efficiency, ratiometric fluorescent sensor, donor concentration, RF in beverages, cytocompatible conjugate CDs-RF

## Abstract

In this work, the Förster resonance energy transfer (FRET) between carbon dots (CDs) as energy donors and riboflavin (RF) as an energy acceptor was optimized and the main parameters that characterize the FRET process were determined. The results were successfully applied in the development of an ultrasensitive ratiometric fluorescent sensor for the selective and sensitive determination of RF in different beverages. Water-soluble CDs with a high quantum yield (54%) were synthesized by a facile and direct microwave-assisted technique. The CDs were characterized by transmission electron microscopy (TEM), Fourier transform infrared spectroscopy (FTIR), dynamic light scattering (DLS), Zeta potential, and UV-visible and molecular fluorescence spectroscopy. The study of the FRET process at two donor concentrations showed that the energy transfer efficiency decreases as the donor concentration increases, confirming its dependence on the acceptor:donor ratio in nanoparticle-based systems. The results show the importance of optimizing the FRET process conditions to improve the corresponding output signal. The variation in the ratiometric signal with the concentration of RF showed linearity in a concentration range of 0 to 11 µM with R^2^ = 0.9973 and a detection limit of 0.025 µM. The developed nanosensor showed good selectivity over other possible types of interference. The sensor was then applied for the determination of RF in beverage samples using the standard addition method with recoveries between 96% and 106%. Preliminary cytocompatibility tests carried out with breast cancer cells (MDA-MB-231) revealed the nanosensor to be cytocompatible in its working concentration regime, even after long incubation times with cells. Altogether, the developed RF determination method was found to be fast, low-cost, highly sensitive, and selective and can be extended to other samples of interest in the biological and food sectors. Moreover, thanks to its long-lasting cytocompatibility, the developed platform can also be envisaged for other applications of biological interest, such as intracellular sensing and staining for live cell microscopy.

## 1. Introduction

Carbon is a black material, insoluble in water, with a weak fluorescence. However, the properties of this material can change dramatically when carbon structures are synthesized on a nanometric scale. In this direction, the best-known carbon nanomaterials include carbon nanotubes, nanodiamonds, carbon nanofibers, fullerene, and graphene and, more recently, quantum dots of carbon or carbon dots (CDs) have attracted interest in many fields [1].

CDs are a type of carbon nanomaterials with sizes less than 10 nm [2]. They have become very popular due to their enhanced photoluminescent properties and excellent photostability. They show a quantum yield (QY) and photochemical stability similar to those of the well-known and commercialized colloidal nanocrystalline semiconductors quantum dots (QDs). Additionally, they are soluble in water, their fluorescence emissions can be modulated with the excitation wavelength, and they have the advantage of being non-toxic materials according to in vivo toxicity tests [1,2,3]. Due to their excellent optical properties, high photostability, simple manufacturing process, low cost, and biocompatibility, CDs have received a great deal of attention for their potential application as sensors and as nanoplatforms for diagnosis and therapy. In particular, their application in Förster resonant energy transfer (FRET)-based systems are of great interest and the subject of exploration. In the FRET process, a non-radiative energy transfer occurs between the donor and the acceptor through dipole–dipole interactions. In FRET systems that involve CDs or other nanoparticles, such as quantum dots (QDs), the interactions are based on the point-dipole model [4,5]. For an energy transfer to occur, the donor and the acceptor must be at a favorable distance (less than 10 nm) [6], and the donor’s fluorescence emission spectrum must overlap the acceptor’s absorption spectrum. This technique is commonly used to determine the distance between two molecules and their specific location, whereas other methods are not sufficiently accurate as compared with FRET. In particular, the use of CDs in FRET-based platforms has great potential in different areas. CDs offer new and versatile opportunities, as they are easily functionalized, facilitate the design of more efficient FRET systems, and provide better analytical characteristics, such as lower detection limits in the quantitative determination of species. In contrast to conventional fluorescent materials such as organic dyes, the peak emission of CDs can easily be controlled over a wide wavelength range, which makes them suitable for detecting various biomolecules, including vitamins. On the other hand, the surface structure of CDs can easily be designed to show a strong attraction to specific molecules, which allows for a very close distance between the CDs and the acceptors [7]. These excellent properties satisfy the requirements of a FRET system and make CDs excellent potential donors with high selectivity and sensitivity.

Riboflavin (RF) is a water-soluble vitamin and is present in many categories of foods. This vitamin plays an essential role in energy production in the body and participates in the metabolism of other vitamins, fats, carbohydrates, and proteins [8]. RF works as an antioxidant, fighting free radicals in the body. Its deficiency causes several human diseases, such as fatigue, slowed growth, and cardiovascular and gastrointestinal problems [9]. It has been reported that RF can also prevent different types of cancers [10].

Accordingly, the determination of RF has become very important and, therefore, different methods have been developed for the determination of RF, including spectrophotometric methods [11], high-performance liquid chromatography [12], electrochemical methods [13,14], and capillary electrophoresis [15]. However, these methods have multiple shortcomings, including a slow response, low selectivity, the use of a large amount of solvent, high-cost equipment, and laborious procedures. Alternatively, methods based on the FRET process using different nanoparticles as a donor have also been reported [16,17,18,19]. In general, these reports do not include a study of the conditions to obtain a FRET process with the highest efficiency. The key difference between the use of dyes and nanoparticles in the FRET process is that, compared with dyes, nanoparticles are larger and their assemblies may be non-homogeneous [20]. Notably, the common FRET theory was developed for molecules and considers the donors and acceptors as dipoles. This theory also states that the FRET efficiency is independent of the donor concentration. However, in nanoparticle-based systems the energy transfer efficiency depends on the acceptor:donor ratio [21]. These aspects show that in FRET systems involving nanoparticles, such as the sensor under study, it is necessary to carry out a systematic study of the interaction between the donor and the acceptor to optimize the conditions under which the highest efficiency of the FRET process can be obtained. The study should include not only optimization to achieve the greatest overlap between the emission spectrum of the donor and the absorption spectrum of the acceptor, but also a study of the influence of the donor concentration on the efficiency of the FRET process.

The structure of RF is pH-dependent. The cationic species present at pH < 0.4 are not fluorescent, while the anionic species present at pH > 9.7 have a weak fluorescence and the neutral species have a fluorescence emission with a high QY [22]. Therefore, the study should be carried out in a neutral environment.

**Scheme 1:**
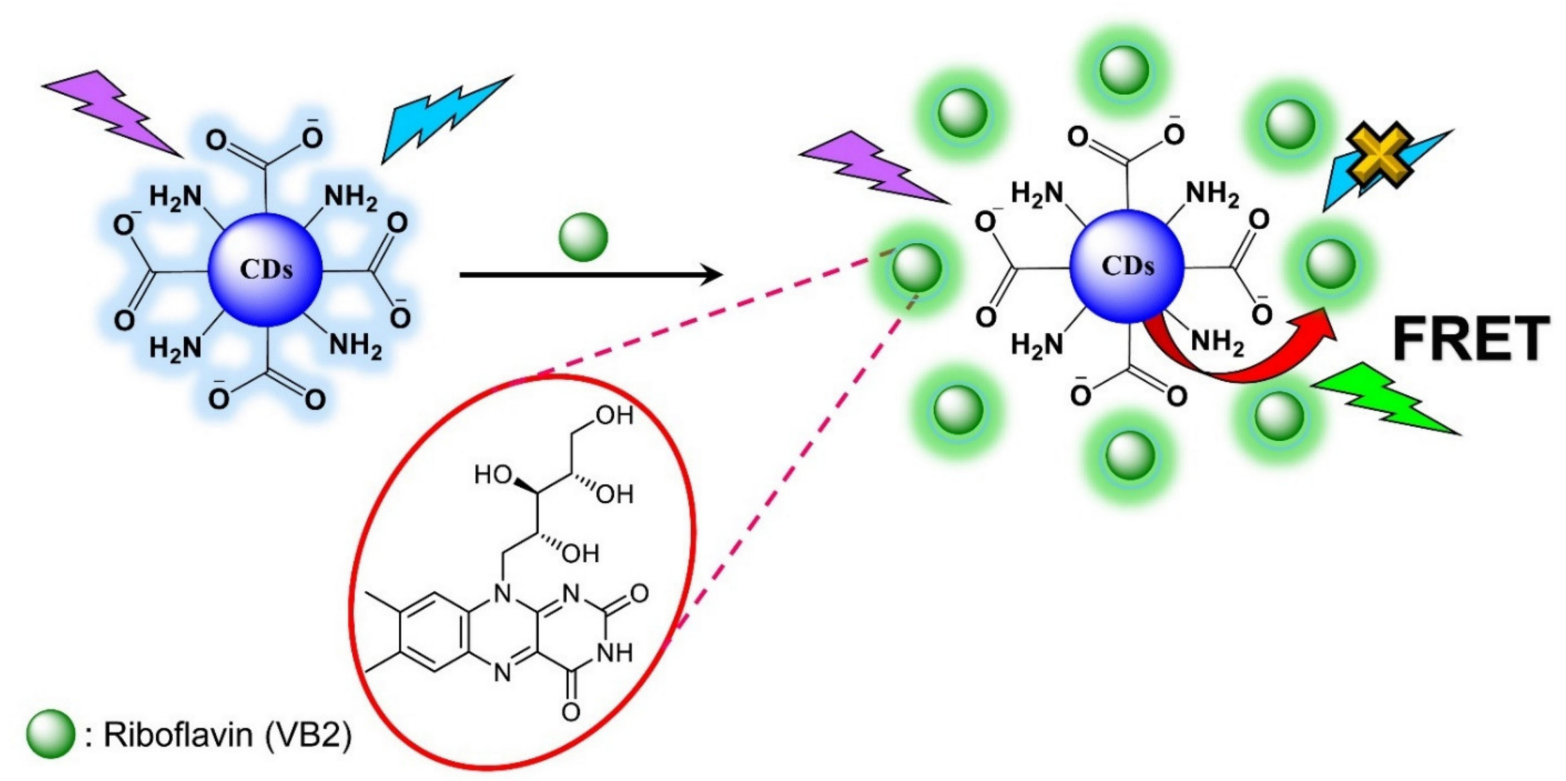
Schematic illustration of the sensor response mechanism.

Considering the great importance of this vitamin, the development of a new, sensitive, precise, easy to perform, and low-cost method for RF determination is very essential. Moreover, the sensing method and probe to be employed have to be mild and cytocompatible. In this context, we herein report the synthesis and characterization of CDs with a high QY and fluorescence emission in the visible light region. The energy transfer process in CDs–RF assemblies as energy donor–acceptor systems is studied and thereby the optimized conditions for obtaining high FRET efficiency were established. In comparison with other methods for RF determination based on the FRET process using nanoparticles, this is the first time that a systematic study to achieve the highest efficiency FRET process has been reported. The key parameters that characterize the FRET process were also determined. Scheme 1 shows a schematic illustration of the working mechanism of the developed nanosensor. This ratiometric sensor presents linearity in a RF concentration range from 0 to 11 µM with a detection limit of 0.025 µM. The nanosensor was successfully applied in the determination of RF in different beverages with excellent results. Importantly, it was also validated as cytocompatible upon its direct administration and long-lasting incubation with cells in culture.

## 2. Materials and Methods

### 2.1. Reagents and Apparatus

The chemicals citric acid (CA, >98%), ethylenediamine (EDA, >99%), quinine hemisulfate salt monohydrate (bioreagent 99–100% yield, quantum yield = 54% in H_2_SO_4_ 0.5 M), ascorbic acid (AA, >99%), riboflavin (RF, >98%), L-lysine (Lys, >99%), L- histidine (His, >98%), L-tryptophan (Trp, >98%), L-asparagine (Asn, >99%), L-glycine (Gly, >99%), L-phenylalanine (Phe, >98%), L-alanine (Ala, >98%), L-glutamine (Gln, >99%), L-arginine (Arg, >98%), L-proline (Pro, >99%), L-glutamic acid (Glu, >98%), glutaraldehyde solution (25 *w*/*v*% in water), crystal violet, orthophosphosric acid, formic acid, 2-N-morpholinoethanesulfonic acid (MES), and phosphate-buffered saline (PBS) were purchased from Sigma-Aldrich, Naucalpan de Juárez, Mexico. Dulbecco’s modified Eagle medium (DMEM), fetal bovine serum (FBS), MEM non-essential amino acids (NEAA; 100×), sodium pyruvate (100 mM), penicillin-streptomycin (pen-strep; 100 mg/mL), and trypsin-EDTA (0.25%, 0.913 mM EDTA) solutions were from Invitrogen. We bought 96-well plates, T-25 cell culture flasks, and disposable sterile filters (pore size: 0.22 μm) from Corning Costar (Glendale, AZ, USA). Other reagents (of analytical grade) and solvents (of spectroscopic grade) were also purchased from Sigma-Aldrich, Naucalpan de Juárez, Mexico.

Absorption spectra were recorded with a Varian Cary-100 UV-visible spectrophotometer (Santa Clara, CA, USA) with a 1 cm quartz cell. Emission spectra were acquired with a Horiba NanoLog fluorescence spectrophotometer (HORIBA Scientific, Edison, NJ, USA) using a xenon lamp as the excitation source equipped with a 1 cm quartz cell. All optical measurements were performed at room temperature under ambient conditions. Transmission electron microscopy (TEM) images were captured using a Jeol JEM2200FS (Akishima, Japan) operated at an accelerating voltage of 200 kV; the microscope has spherical aberration correction in the STEM mode. Zeta potential and Dynamic Light Scattering analyses were performed with a Malvern Panalytical México Zetasizer Nano ZS 3600(Nuevo Léon, México). All pH measurements were made with a Thermo Scientific pH meter (Waltham, MA, USA). Absorption readouts from cytocompatibility tests were acquired with a Microplate Absorbance Reader (iMARK^TM^, BIORAD, Hercules, CA, USA) operating at 595 nm.

### 2.2. Preparation of Carbon Dots (CDs)

A microwave-assisted synthesis method was used to obtain the CDs, according to a previous report [23], but with some changes. In this procedure, CA (1.0507 g) and EDA (335 µL) were dissolved in deionized water (DI-water) (10 mL). The mixture solution was heated (180 °C) in a Microwave Digestion Systems Milestone Start-D (CDMX, Mexico) (power: 1000 W) for 15 min. After cooling to room temperature, the product, which was yellow and transparent, was filtered through a 0.2 mm filtration membrane. The filtered solution was further purified by dialysis in ultra-pure water. Finally, a clear and yellow aqueous solution was freeze-dried to obtain the dry CDs.

The relative QY of CDs was examined by comparison with the fluorescence emission of quinine sulfate using Equation (1):

(1) $$\phi_{x}=\phi_{ST}\left(\frac{Grad_{x}}{Grad_{ST}}\right)\left(\frac{\eta_{x}^{2}}{\eta_{ST}^{2}}\right)$$

where *ST* and *x* refer to the standard of reference and the sample of interest, respectively, *φ* is the fluorescence quantum yield, *Grad* is the gradient obtained from the plot of integrated fluorescence intensity vs absorbance, and *η* is the solvent refractive index. Quinine sulfate was used as the reference standard and has a QY of 54% in H_2_SO_4_ solution (0.1 M).

### 2.3. Investigation of the CDs’ Stability

CDs dispersed in ultrapure water at pH 7.4 in PBS were exposed to UV light (365 nm) for 1 h using a xenon lamp with 450 W of power. The photochemical stability of the CDs was investigated by measuring the fluorescence intensity using the kinetic system of the Nanolog spectrofluorimeter.

### 2.4. FRET Assays between CDs and RF

#### 2.4.1. Selection of the Donor Excitation Wavelength

The selection of the excitation wavelength of the donor (CDs) was made according to the overlap of its emission spectrum with the absorption spectrum of the acceptor (RF). A solution of CDs (4 × 10^−2^ mg/mL) and a solution of RF (10 µM) were prepared in phosphate buffer at pH 7.4 (3 mM). The CDs solution was excited at different wavelengths (320 nm, 340 nm, and 360 nm) and the fluorescence spectrum was recorded. The absorption spectrum of the RF solution was obtained. The overlapping region between the emission fluorescence spectra of CDs and the absorption spectrum of RF was measured. The excitation wavelength of the CDs was selected according to the maximum overlap between the donor emission spectrum and the acceptor absorption spectrum.

#### 2.4.2. Selection of the Donor Concentration

Titrations of the CDs solutions were carried out as follows. A 3 mL aliquot of the corresponding donor solution (4 × 10^−2^ mg/mL and 2.5 × 10^−2^ mg/mL) was added to a quartz cell. Successive amounts (20 µL) of the RF solution (1000 µM) were added under constant stirring until a RF concentration of 63 µM was reached. The starting solution of the CDs and the RF titrant solution were prepared in phosphate buffer at pH 7.4 (3 mM). The resulting fluorescence spectrum was obtained in each addition at an excitation wavelength of 360 nm.

#### 2.4.3. Calculation of FRET Parameters

FRET is a non-radiative energy transfer that occurs via dipole–dipole interactions, from an excited donor molecule to an acceptor molecule, which can return to the ground state by emitting a photon of lower energy [21]. The efficiency of the FRET process can be calculated through Equations (2)–(5). Efficiency can be measured experimentally and is frequently expressed as follows:

(2) $$E=1-\frac{F_{DA}}{F_{D}}=\frac{R_{0}^{6}}{R_{0}^{6}+r^{6}}$$

where *F_DA_* is the donor integrated fluorescence (CDs) in the presence of the acceptor (RF) and *F_D_* is the donor integrated fluorescence alone. In this Equation, *R*_0_ is the Förster distance, which is the separation distance that yields a 50% energy transfer efficiency and *r* is the distance between the donor and the acceptor.

In our case, because CDs have large surfaces that present multiple binding sites, a single nanoparticle can simultaneously interact with several proximal acceptors. In a such configuration, the above efficiency can be expressed as [21]:

(3) $$E=\frac{nR_{0}^{6}}{nR_{0}^{6}+r^{6}}$$

where *n* is the total number of acceptors interacting with the same donor.

The Förster distance (*R*_0_*)* is expressed as:

(4) $$\;R_{0}^{6}=8.79\times 10^{-25}K^{2}\eta^{-4}\phi J\left(\lambda \right)$$

where the dipole orientation factor *K^2^* and the refractive index *η* are accepted as 2/3 and 1.336, respectively [24], *ϕ* is the fluorescence quantum yield of CDs in the absence of RF, and *J(λ)* is the integral of the spectral overlap, which can be calculated from the following equation:

(5) $$J\left(\lambda \right)=\int_{0}^{\infty }F_{D}\left(\lambda \right)\varepsilon_{A}\left(\lambda \right)\lambda^{4}d\lambda$$

where *F_D_(λ)* is the fluorescence intensity of the fluorescence donor at a given wavelength, and *ε_A_ (λ)* is the molar absorptivity of the acceptor at a given wavelength.

### 2.5. Interference and Competition Analysis

The influence of different L-amino acids (Lys, Ala, Gly, Trp, Phe, Glu, Gln, Arg, Pro, His, and Asn) and ascorbic acid (AA) on the FRET nanoprobe response was studied through fluorescence spectra. For the interference experiment, 2 mL of CDs solution (5 µg/mL) and 700 µL of each possible interference solution (100 µM), both in phosphate buffer (3 mM, pH 7.4), were added to a fluorescence cell with a 1 cm optical path. The fluorescence spectrum of CDs was obtained after the addition of each interference solution under an excitation wavelength of 360 nm. The competition analysis was conducted by measuring the fluorescence of the CDs in the presence of RF and the coexisting substance under the same conditions as the interference experiment. A solution of CDs–RF conjugate was prepared by mixing 2 mL of CDs solution (5 µg/mL) and 700 μL of RF solution (100 µM) in phosphate buffer (3 mM, pH 7.4). The conjugate solution was placed in a quartz fluorescence cuvette with a 1 cm optical path. Each possible interference solution was added (700 µL, 100 µM) and the fluorescence spectrum was obtained under an excitation wavelength of 360 nm.

### 2.6. Cell Culture

MDA-MB-231 breast cancer cells were cultured in DMEM supplemented with 10% FBS, 0.1 mM NEAA, 1 mM sodium pyruvate, and 1% pen-strep and incubated under standard culture conditions (37 °C in a humidified atmosphere with 5% CO_2_) within T-25 cell culture flasks. Cells were passaged when they reached an 80−90% optical confluence. Confluent monolayers were then treated with the trypsin-EDTA solution and incubated for 4 min under culture conditions for detachment. Cells were then pelleted, resuspended in culture medium, and seeded in 96-well plates as required. Sterile filtered Milli-Q water was used throughout. All experiments were carried out under sterile conditions. The MDA-MB-231 cell line was purchased from the American Type Culture Collection (ATCC).

### 2.7. Cytocompatibility Test

CDs and RF were dissolved separately in 10 mM PBS (1× PBS) to produce stock solutions of 10 mg/mL and 7.44 × 10^−3^ M, respectively. These stock solutions were serially diluted (also in 1× PBS) and mixed as necessary to obtain the samples to be tested. The final CDs samples were diluted to concentrations in the range of 0.01–5 mg/mL, while the CDs–RF conjugate was prepared upon the 1:1 mixing of CDs (0.01 mg/mL) and RF (7.44 × 10^−5^ M) solutions. The cytocompatibility of the prepared samples was determined by the well-known crystal violet staining method [25,26,27,28]. To this end, the MDA-MB-231 cells were seeded in 96-well plates (100 μL, 1.5 × 10^4^ cells/well) and grown for 24 h under standard culture conditions in DMEM supplemented with 10% FBS, 1% pen-strep, 1 mM sodium pyruvate, and 0.1 mM NEAA. Afterwards, the samples were internalized (100 μL) and incubated for the chosen period (24 h and 48 h). Once the desired incubation times had elapsed, the culture medium was discarded and the cells were shaken at room temperature (300 rpm, 15 min) in the presence of 10 μL of a glutaraldehyde solution (11 *w*/*v*% in water). The solution was discarded and cells were washed two times with Milli-Q water. The cells were then shaken at room temperature (300 rpm, 15 min) in the presence of 100 μL of a crystal violet solution (0.1 *w*/*v*% in 200 mM orthophosphoric acid, 200 mM formic acid, and 200 mM MES, pH 6). The solution was discarded, and the cells were again washed twice with Milli-Q water. Once washed, the cells were incubated at room temperature overnight for drying. Once dried, the cells were shaken at room temperature (300 rpm, 15 min) in the presence of 100 μL of acetic acid (10 *w*/*w*% in water). Immediately after, the absorbance of the resulting solution was measured at 595 nm. The percentage of cell viability was quantified as:

(6) $$\%\;Cell\;viability=100*\frac{A_{S}}{A_{C}}$$

where *A_S_* and *A_C_* stand for the absorbance of the treated (exposed to the samples) and control (non-exposed) cells, respectively. The results are the average of six independent experiments. Sterile filtered Milli-Q water was used throughout. Sterile conditions were preserved up until cell fixation with glutaraldehyde.

### 2.8. Statistical Analysis

Statistical analysis was performed by the one-way analysis of variance (ANOVA). The post hoc Bonferroni test was used to perform multiple comparisons and differences were considered significant at a level of *p* < 0.05.

## 3. Results and Discussion

### 3.1. Synthesis and Characterization of CDs

**Figure 1:**
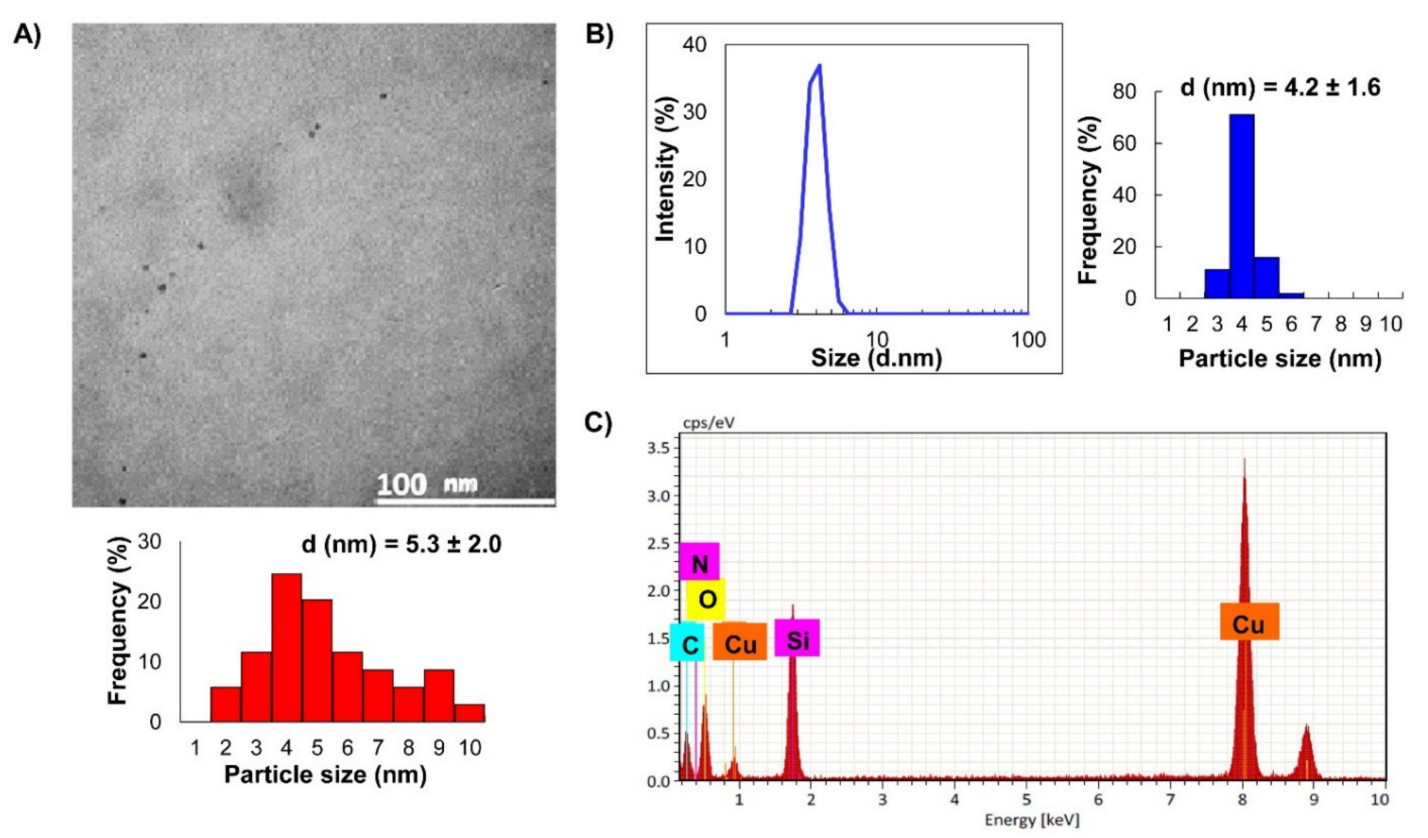
(**A**) Scanning transmission electron microscopy (STEM) image (top) and the size distribution histogram (bottom), (**B**) Size distribution of CDs and the corresponding size distribution histogram obtained by DLS, and (**C**) EDS results for CDs.

CDs were obtained by a microwave-assisted method using CA and EDA in an aqueous medium. The solution of CDs shows a homogeneous phase with no evidence of aggregations and good water dispersibility. This behavior is consistent with a Zeta potential of −22 mV (Figure S1). The TEM image of the prepared CDs (Figure 1A, top) indicates uniformly dispersed spherical nanoparticles, and the size distribution histogram obtained from different enlarged images shows a diameter of around 5.2 ± 2.0 nm (Figure 1A, bottom). The corresponding size distribution histogram obtained by dynamic light scattering (DLS) (Figure 1B) illustrates that the size of the CDs lies in the range of 3.6–5.6 nm with an average size of 4.2 ± 1.6 nm. This result agrees with that obtained by TEM. The composition of the CDs was determined using EDS analysis. Figure 1C shows the characteristic peaks of C, O, and N. The other observed signals correspond to the elements present in the sample holder. The stability of the CDs was also studied by irradiating the sample dispersed in water at pH 7.4 under a xenon lamp at 365 nm for 1 h. The results show the high optical stability of the CDs (Figure S2).

**Figure 2:**
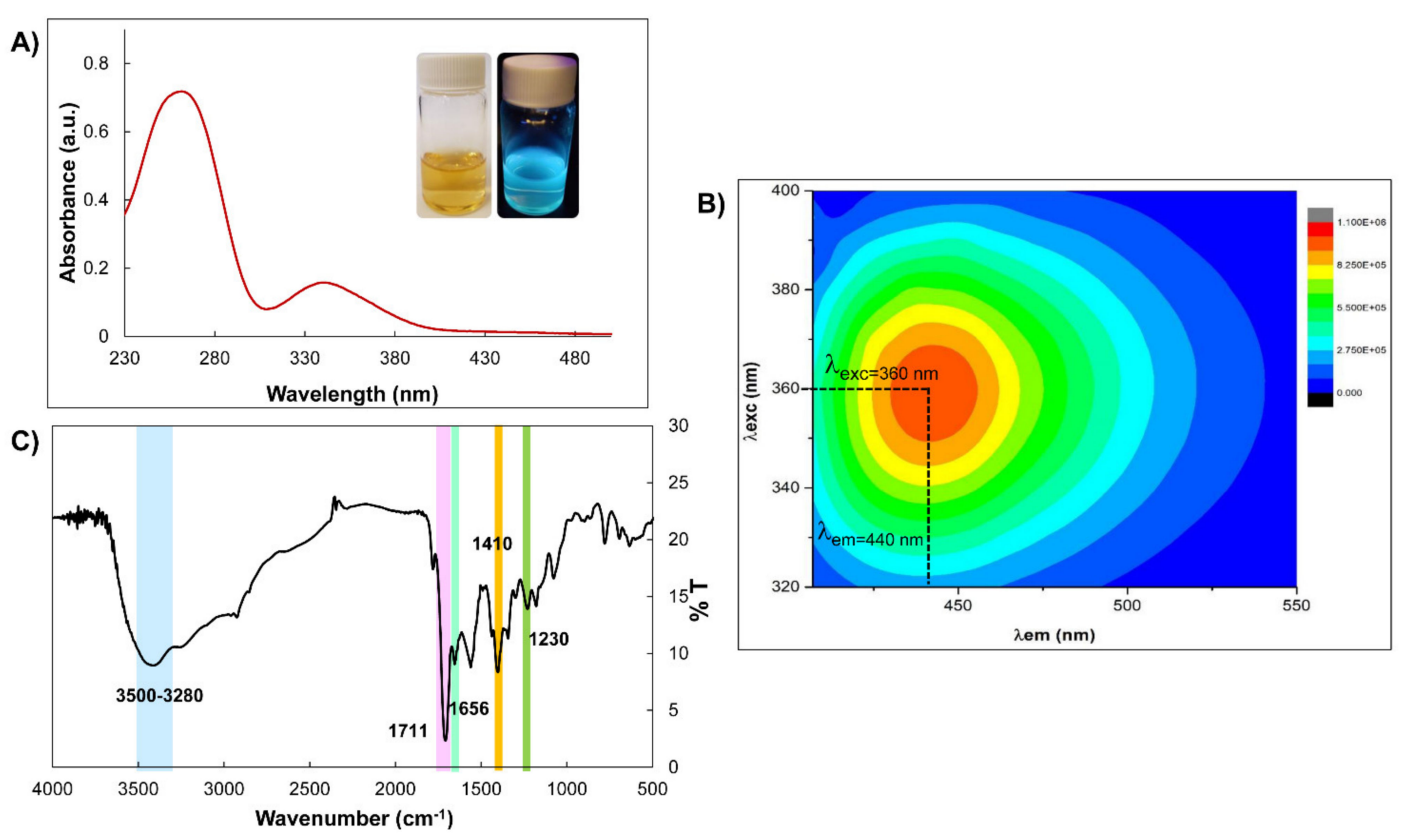
(**A**) Absorption spectra of CDs (inset: CDs under white (left) and UV light (right)), (**B**) 2D-contour diagram of excitation/emission spectra of CDs, and (**C**) FT−IR spectrum of CDs.

CDs are made up of an amorphous or a crystalline core, wherein sp^2^ carbon predominates, and of an oxidized carbon surface with the presence of carboxyl and hydroxyl groups [29]. CDs were characterized by UV-visible absorption and fluorescence spectroscopy. Figure 2 shows the absorption and fluorescence spectra of the CDs solution. As can be observed in Figure 2A, the UV-visible spectrum of CDs shows a strong absorption band at 261 nm attributed to the π–π* electronic transitions corresponding to the C=C groups and a second weaker absorption band at 340 nm attributed to *n*–π* electronic transitions corresponding to the C=O groups in the structure. The inset in Figure 2A shows photographs of CDs under white (left) and UV light (right). Figure 2B shows the corresponding 2D-contour diagram of excitation/emission spectra of CDs, which exhibit an excitation-dependent emission character. It should be noted that the emission fluorescence band is shifted to a longer wavelength as the excitation wavelength increases from 300 to 460 nm, which is a typical characteristic of CDs. This behavior has been attributed to the presence of CDs with different sizes and different surface states due to the organic groups present on the surface of CDs [30]. The strongest emission fluorescence band was obtained at the excitation wavelength of 360 nm. The QY of CDs was determined to be 54%, indicating their excellent fluorescence properties.

CDs were further analyzed by FT-IR spectroscopy to identify the presence of functional groups. The corresponding spectrum is shown in Figure 2C. The broad band in the region of 3500 and 3280 cm^−1^ is attributed to the overlapping of stretching vibrations of the O–H and N–H bonds. The small band at 2890 cm^−1^ corresponds to the stretching vibrations of C–H bonds. The peaks at 1711 and 1410 cm^−1^ indicate the presence of COO^−^ functional groups. The band at 1656 cm^−1^ corresponds to the stretching vibrations of C=C bonds, the band at 1590 cm^−1^ corresponds to the flexion vibrations of N-H bonds, and the band at 1230 cm^−1^ corresponds to the stretching vibration of C–N bonds. These results demonstrate the presence of carboxyl, N–H, and C–N groups on the surface of the as-synthesized CDs, which are due to the use of CA and EDA in the synthesis. The presence of these groups on the surface of the CDs shows the possibility of H-bonding with the functional groups of the RF, decreasing the spatial distance between the CDs and RF and favoring the FRET process.

### 3.2. Optimization of the FRET Process

#### 3.2.1. Selection of pH

First, the optimum pH for an effective FRET process was investigated. The Zeta potential of the CDs was measured at different pH values (from 3 to 10, Figure S1). The results reveal an evolution in the Zeta potential of the CDs from positive values at a low pH (3–4) to negative values at higher ones (6–10), having a pH value of around 4.4 as the isoelectric point. The positive charges at low pH values can be ascribed to the presence of protonated amine groups along the surface of the CDs. On the other hand, the isoelectric point, around pH 4.4, is consistent with the pK_a_ of the carboxyl groups also present along the surface of the CDs. Finally, the highest negative Zeta potential values obtained between pH 6 and 10 can be ascribed to a dominating contribution of the carboxylate groups at these higher pH values. Under these conditions, CDs are stable and display high dispersibility in water. In addition, hydrogen bonds are expected to occur between RF and the COO^−^ and amine groups present on the surface of CDs. Based on these findings, and aiming to work under physiological conditions, pH 7.4 was selected as the pH of interest. Moreover, the neutral species of RF have a fluorescence emission with a high QY at this pH [22].

#### 3.2.2. Selection of the Excitation Wavelength

**Figure 3:**
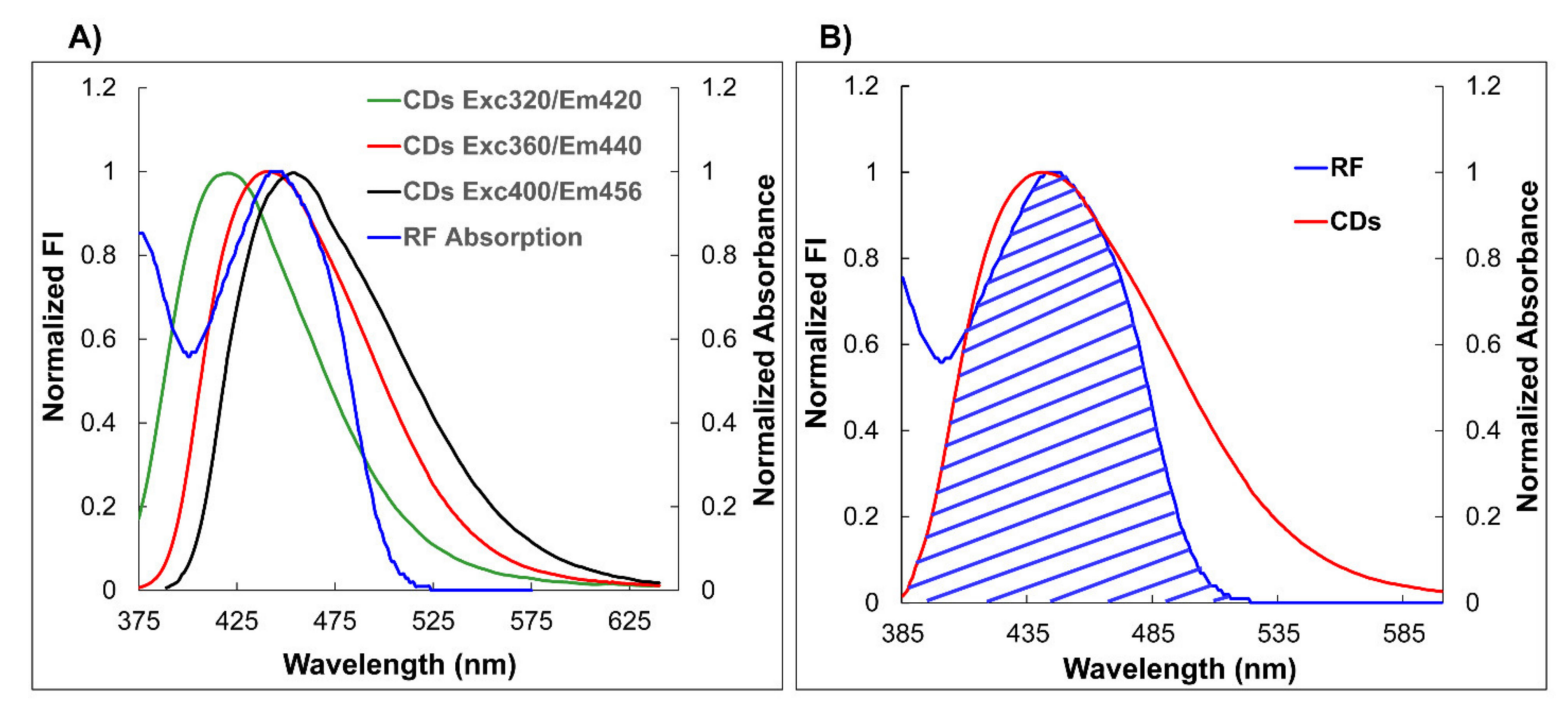
(**A**) UV-visible absorption spectrum of RF and emission spectra of CDs at different excitation wavelengths and (**B**) Spectral overlap between the emission spectrum of CDs (Exc 360/Em 440) and the RF absorption spectrum.

A factor that influences the efficiency of a FRET process is the overlap between the donor emission spectrum and the acceptor absorption spectrum. To analyze the possibility of the occurrence of the FRET process between CDs and RF, the region of overlap between the emission fluorescence spectra of CDs and the absorption spectrum of RF was obtained. The absorption spectrum of RF and the emission spectra of CDs excited at different wavelengths are shown in Figure 3A. As can be seen, the maximum emission peaks of the CDs are at 420, 440, and 456 nm when the CDs were excited at 320, 340, and 360 nm, respectively, while the maximum absorption of RF was observed at 445 nm. The emission spectrum of CDs excited at 360 nm (maximum peak at 456 nm) shows the largest spectral overlap with the absorption spectrum of RF (Figure 3B), which indicates the possibility of the FRET process from CDs to RF. In addition, as mentioned above, at this excitation wavelength, the highest QY of CDs was obtained. Accordingly, this excitation wavelength was selected for the FRET process. The possible influence of direct RF excitation on the FRET process’s efficiency is discussed in the next section.

#### 3.2.3. Influence of Donor Concentration

As has been mentioned before, there are differences between dyes and nanoparticles in the FRET process. The main differences arise from the bigger size of nanoparticles and the somewhat heterogeneous nature of nanoparticle assemblies [20]. The common FRET theory was developed for the molecules, and it considers donors and acceptors as dipoles. This theory also states that the FRET efficiency is independent of the donor concentration for molecule pairs. Since our FRET platform involves nanoparticles of CDs, it was necessary to study the influence of donor concentration on the efficiency of the energy transfer.

**Figure 4:**
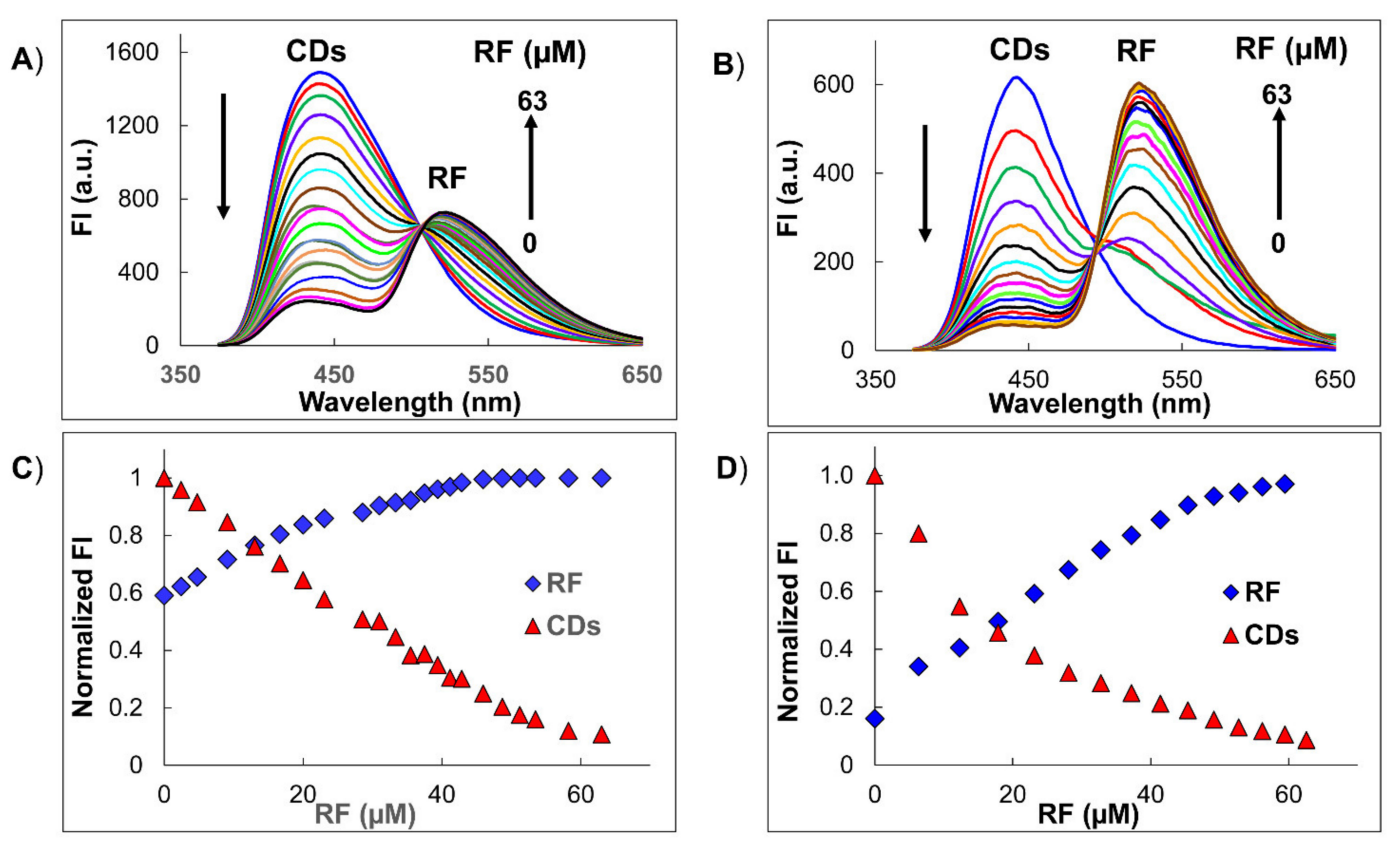
Spectra of the successive titration of CDs with RF at two donor concentrations (**A**) 4.0 × 10^−2^ mg/mL and (**B**) 5.0 × 10^−3^ mg/mL and the normalized fluorescence intensity signal versus RF at two donor concentrations (**C**) 4.0 × 10^−2^ mg/mL and (**D**) 5.0 × 10^−3^ mg/mL. The RF concentrations range from 0 µM to 63 µM in all experiments.

The successive titrations of CDs with RF at two different donor concentrations are shown in Figure 4A,B. The titration spectra show a progressive decrease in the donor fluorescence and an increase in the acceptor fluorescence, evidencing the occurrence of the FRET process. However, the system with a lower donor concentration shows a donor fluorescence quenching and an acceptor fluorescence enhancement greater than those in the system with a higher donor concentration. Namely, the efficiency of the FRET process decreases with the increase in donor concentration. The efficiency (*E*) of both systems was calculated using Equation (2) after the deconvolution of the corresponding spectra. For the system shown in Figure 4A (donor concentration = 4.0 × 10^−2^ mg/mL), the efficiency (*E*) was 84% and for the system shown in Figure 4B (donor concentration = 5.0 × 10^−3^ mg/mL), the efficiency (*E*) was 91%. The plots of normalized fluorescence intensity signal versus RF concentration at two donor concentrations are shown in Figure 4C,D. The graphs indicate that at a higher donor concentration (Figure 4C), the saturation is reached at a lower RF concentration, with an acceptor fluorescence enhancement of only 40%. Interestingly, when an excess of RF is added in both titrations (Figure 4A,B), the fluorescence intensity remains practically constant, indicating that under these conditions the direct emission of RF is not considerable.

The enhancement in FRET efficiency with increasing dye-to-nanoparticle ratio has been previously reported [21,31]. The theory that explains the energy transfer between conventional organic molecules is based on dipole–dipole interactions and may not apply to pairs involving nanoparticles such as the system under study (CDs–RF). One of the reasons is that nanoparticles are much larger in size than conventional organic fluorophores and have a large surface area that presents multiple binding sites. Then, a single nanoparticle can interact with several proximal acceptors and act as a central energy donor. Such a configuration produces an increase in the overlap integral proportional to the increase in the number of acceptors around the nanoparticle. Consequently, there is an improvement in the overall energy transfer efficiency compared with a one-to-one configuration. Due to this behavior, the study of the influence of the donor concentration, which affects the acceptor:donor ratio, for a constant acceptor concentration is very important to achieve a good efficiency of the FRET process and to obtain good analytical characteristics of the sensor. At a higher donor concentration (Figure 4A), there are simply less RF acceptors per CD donor and, thus, a lower FRET efficiency, while at a lower donor concentration (Figure 4B) there are more RF acceptors per CD donor and, consequently, a higher FRET efficiency. Other authors have reported that the change in efficiency with the donor concentration is also influenced by the competition that exists between the donor and the acceptor and the donor and the donor in an energy transfer [32].

The FRET parameters for the system with better efficiency were calculated using Equations (2)–(5). Table 1 shows the overlap integrals (*J*), the FRET efficiency (*E*), Förster distances (*R*_0_), the distance between the donor and the acceptor (*r*), and the binding constant (*K*). The binding constant was calculated using the modified Stern–Volmer equation (Supplementary Materials). The value of *K* is 2.7 × 10^4^ M^−1^ with a correlation coefficient of 0.9937 (Figure S4B). The binding constant, which is greater than 10^4^ M^−1^, suggests that there is a moderate interaction of CDs with RF. In our case, the *K* value acquires more relevance since it was obtained in an aqueous competitive medium. Then, the value of *K* shows the high affinity of RF for CDs. This is an important result that influences the sensitivity and selectivity of the sensor. The energy transfer process between CDs and RF was confirmed by the *R*_0_ and *r* values in the interval of 2 nm to 8 nm, and the fulfillment of the required condition: 0.5*R*_0_ < *r* < 1.5*R*_0_ [33].

### 3.3. Calibration Curve

**Figure 5:**
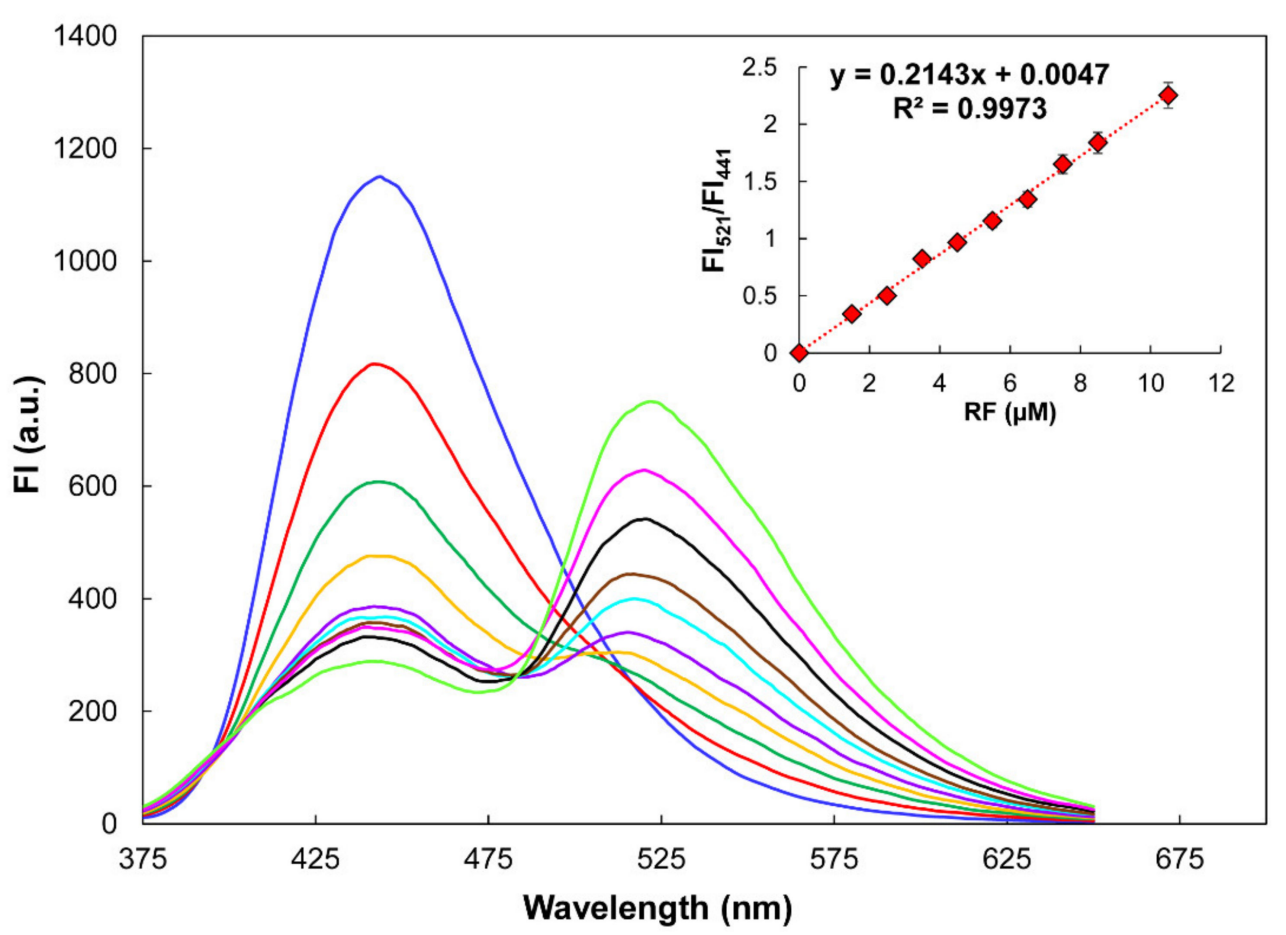
Variation in the fluorescence emission of the CDs–RF conjugate in the presence of different RF concentrations (0 µM to 11 µM). Inset: linear relationship between the fluorescence emission ratio (I_521_/I_441_) of the CDs–RF conjugate and the RF concentration.

The calibration curve was obtained through the ratiometric measurement (I_521_/I_441_) of the RF fluorescence emission enhancement (λ = 521 nm) and the CDs fluorescence emission quenching (λ = 441 nm) at different RF concentrations. Figure 5 shows the variation in fluorescence spectra of CDs–RF conjugates in the presence of various RF concentrations. The inset of Figure 5 shows the RF calibration curve (average of three repetitions), with a linear concentration range from 0 to 11 µM, a correlation coefficient of 0.9973, and a detection limit (DL) of 0.025 µM. The DL of RF in CDs was calculated using the formula DL = 3σ blank/S, where σ is the standard deviation of the blank signal (*n* = 10) and S is the slope of the calibration curve. The ratiometric signal of the fluorescence intensities provides a very stable and reproducible measurement that eliminates the possible errors of the determination due to fluctuations in the source, photobleaching, background, etc.

### 3.4. Selectivity of CDs–RF Conjugates for the RF Detection

**Figure 6:**
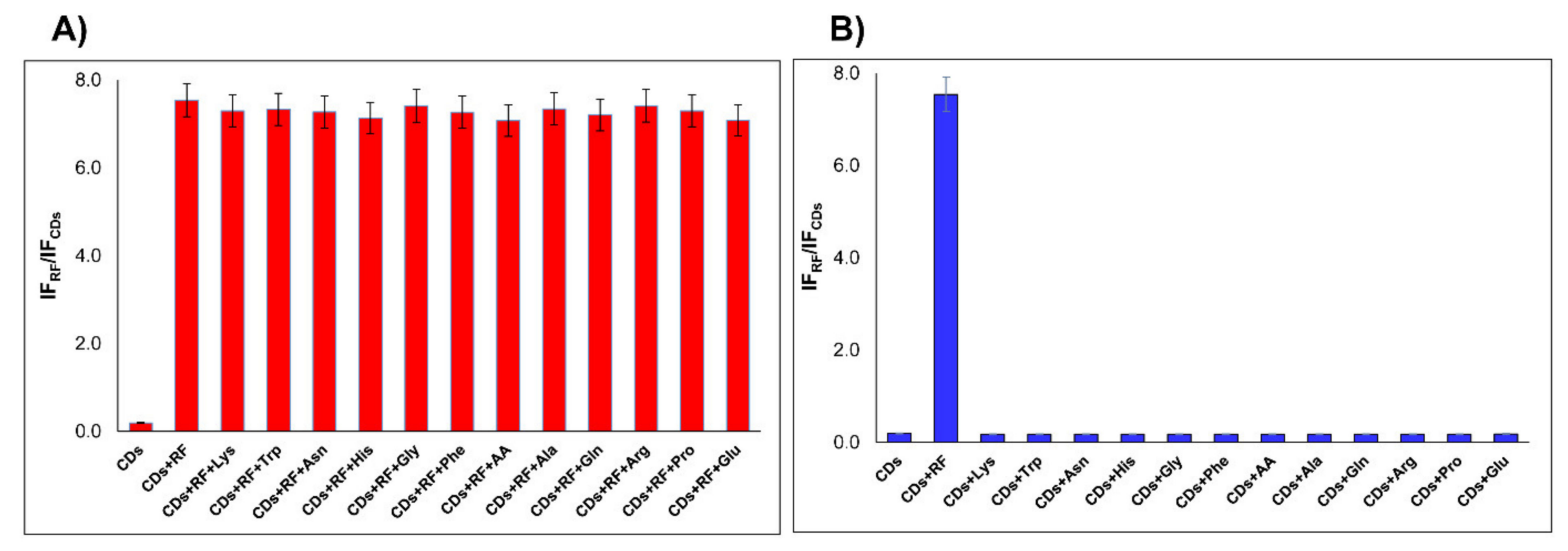
(**A**) Fluorescence emission ratio (I_521_/I_441_) of CDs–potential interferences and (**B**) fluorescence emission ratio (I_521_/I_441_) of CDs–RF–potential interferences (molar ratio of RF:interference = 1:1). PBS was used throughout (pH = 7.4).

The influence of possible interfering substances that are commonly present in biological or food samples was also examined. Different representative substances, including L-amino acids (Lys, Trp, Asn, His, Gly, Phe, Ala, Gln, Arg, Pro, and Glu) and ascorbic acid (AA), were analyzed at pH 7.4. At physiological pH, the CDs are negatively charged (Figure S1) and the pH is above the isoelectric point of RF (pI = 6). Under these conditions, electrostatic interactions are not expected. However, all the interferences studied present groups that can form hydrogen bonds with the active groups on the surface of the nanoparticles. As can be seen in Figure 6A, only the CDs–RF signal showed a remarkable output, while the rest of the substances showed no significant fluorescence intensity variations. The selectivity of the CDs for RF is based on the energy transfer, which occurs only between these two moieties. The study was also carried out through competitive experiments in PBS solution at pH 7.4. The molar ratio of the RF:interfering substance was kept constant at 1:1. As can be seen in Figure 6B, the ratiometric measurement I_521_/I_441_ in the presence of RF and the corresponding interference showed a selective effect by RF over other substances, including L-amino acids (Lys, Trp, Asn, His, Gly, Phe, Ala, Gln, Arg, Pro, and Glu) and AA. These possible interferences in concentrations up to 20 µM do not affect the formation of the CDs–RF conjugate and do not interfere with the ratiometric fluorescence intensity of the nanoprobe. Therefore, the proposed nanosensor demonstrates high selectivity for RF determination.

### 3.5. Comparison with Other Methods

The proposed sensor was also compared with other reported methods for the determination of RF. Methods based on simple UV-Vis absorption or fluorescence spectroscopy show less sensitivity than the proposed method [34,35]. In general, UV-Visible spectrophotometric methods have less sensitivity than spectrofluorimetric methods. Table 2 shows the detection limit and linear range of different methods for RF determination. Our method exhibits good sensitivity towards RF detection without the need for tedious off-line preparations and expensive equipment. Although there are some methods based on the FRET process that use different nanoparticles as donors, this is the first time that a systematic study of the best-optimized conditions to improve the efficiency of the FRET process has been reported. Comparison with these other methods indicates that our nanoprobe has a lower detection limit than most of those reported methods with a good linear range. We consider that the optimization of the FRET process directly influences the analytical characteristics, resulting in a greater sensitivity, selectivity, and linear range. The nanoprobe provides a simple approach to creating a ratiometric fluorescent sensor based on the FRET process for the determination of RF in aqueous media.

### 3.6. The Fluorescent Ratiometric Detection of RF in Real Samples

Three types of beverages (an energy drink, green tea, and wine) were selected to confirm the applicability of this fluorescent ratiometric probe in the determination of RF. The proposed sensor was applied to detect RF directly without previous treatment of the samples using the standard addition method. The results of the sample analysis are given in Table 3. The recovery tests were conducted by adding seven different amounts of RF to each sample. As indicated in Table 3, a good agreement was obtained between the added and found amounts of RF in spiked samples of the energy drink, green tea, and wine, with good recovery percentages (between 96% and 106%) and a relative standard deviation (RSD) lower than 4%, indicating the reliability of this FRET fluorescent probe for the detection of RF. The concentrations of RF in these three analyzed samples are in the same concentration range as reported in previous works on the estimation of RF concentrations [40,41]. The results are the mean of three parallel analyses.

### 3.7. Preliminary Tests of Cytocompatibility

**Figure 7:**
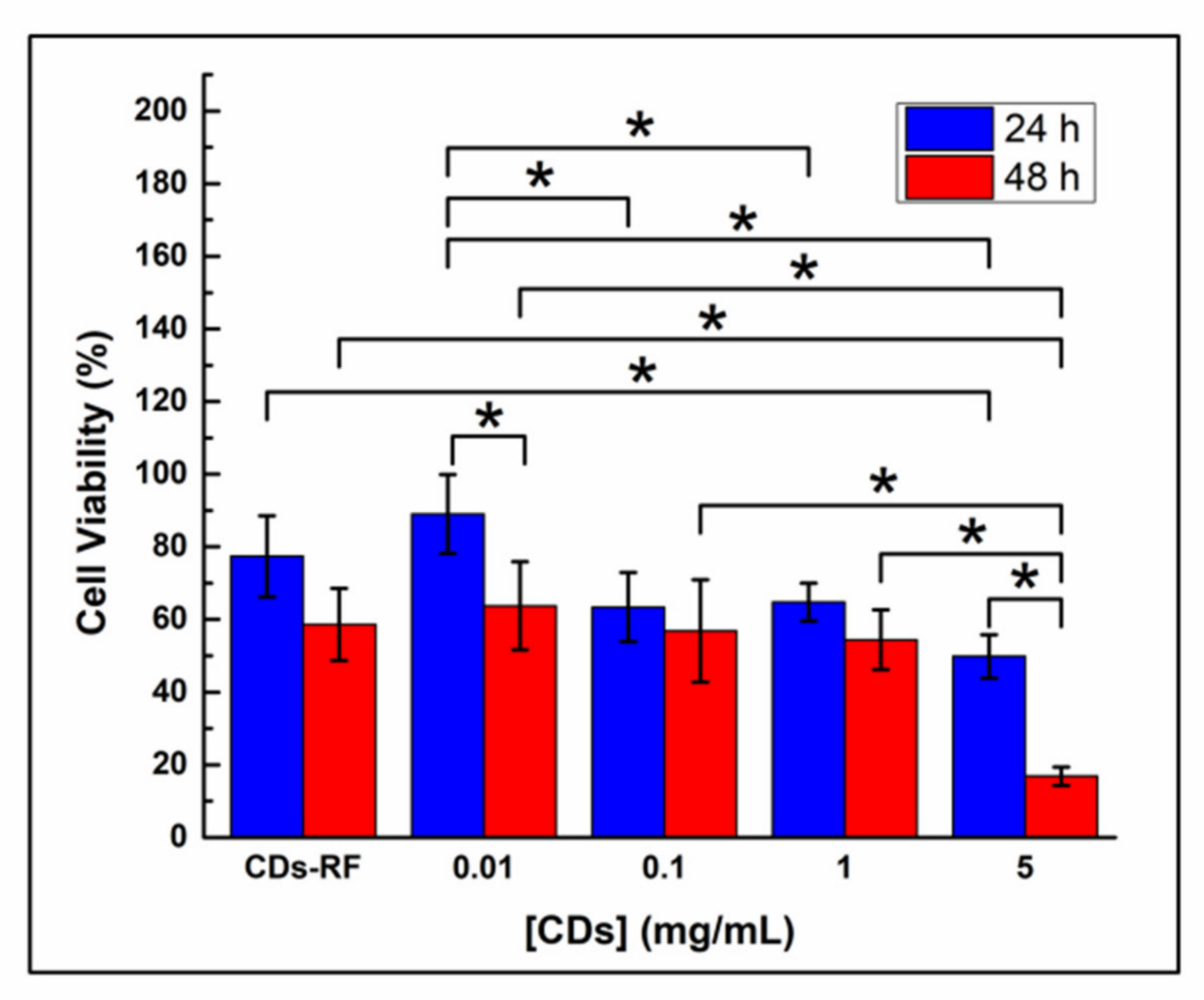
Viability of breast cancer cells (MDA-MB-231) after exposure to CDs at varying concentrations and a CDs–RF conjugate for 24 and 48 h. All samples were dissolved in PBS. The CDs–RF conjugate was prepared upon the 1:1 mixing of CDs (0.01 mg/mL) and RF (7.44 × 10^−5^ M) solutions. (The concentration of the CDs–RF conjugate was calculated this way to match the conditions of the sensing experiments at the resulting 2×-dilution after administration to cells). Statistically significant difference between data (*p* < 0.05) is denoted with *.

Preliminary tests of cytocompatibility were carried out upon the direct exposure of CDs at varying concentrations and a selected CDs–RF conjugate to MDA-MB 231 cells in culture. Aiming to obtain a comprehensive piece of information, the upper concentrations of CDs and their incubation time with cells were extended to higher values as compared to other publications [42,43,44,45,46] and those explored in the previous characterizations (up to 5 mg/mL and 48 h, respectively). Figure 7 shows the viability of the cultured cells at 24 h and 48 h post-exposure. It can be seen from this figure that the CDs show a dose-dependent cytocompatibility at both time points, which is typical of nanomaterials [46]. Analyzing the samples at 24 h first, it is observed that the CDs at the lowest concentration (0.01 mg/mL) along with the CDs–RF conjugate gave rise to outstanding levels of cell viability (ca. 90% and 80%, respectively) without a significant statistical difference among them (*p* > 0.05). According to the five-point scale commonly used to categorize biomaterials upon contact with cells, both samples can be considered to be cytocompatible insofar as the viability values they led to (in the range of 70–90%) correspond to a mild reactivity [28,47,48]. Meanwhile, the rest of the CDs samples (0.1–5 mg/mL) were found to produce slightly lower cell viability values in the range of 50–65%, which correspond to a moderate reactivity [47,48]. With respect to the samples incubated for 48 h, on the other hand, both the CDs–RF conjugate and the CDs samples in the range of 0.01–1 mg/mL also reflected a moderate reactivity [47,48], leading to cell viability values of ca. 55–60%. By contrast, the most concentrated CDs sample (5 mg/mL) rendered a cell viability of ca. 17%, which corresponds to a severe reactivity [47,48]. As already mentioned, it is worth noting that the experimental procedure here followed entailed the incubation of the samples for the whole length of the experiments (24 h and 48 h), which might have contributed to the moderate-to-severe reactivity displayed by the concentrated sample at 48 h. Other publications in the field have considered the exchange of cell culture medium at short periods after the administration of the respective nanomaterials [43,44,45,46], which might help to reduce probable scenarios of cytotoxicity. Whatever the case, it is remarkable that the CDs herein produced proved to be cytocompatible at the concentration regime at which they are used as sensors of RF. Moreover, RF is not expected to confer any cytotoxicity on the CDs–RF conjugate given that it has been demonstrated to be totally cytocompatible at concentrations up to 5 mM (a concentration ca. two orders of magnitude higher than the one here studied) [49]. In addition to the mild synthesis process, herein optimized, the observed cytocompatibility of the CDs and the CDs–RF conjugate can certainly be related to the natural occurrence of RF in the organism and the proper solubility of both (CDs and RF) in PBS. The outstanding cytocompatibility of the developed platform under its working conditions, even after a long incubation time with cells, makes us envisage it as highly attractive for other applications in the biological field, such as intracellular sensing and staining for live cell microscopy [50]. In particular, the CDs–RF probe can be tested to study the transport and intracellular localization of RF, similar to other probes based on the FRET process [51].

## 4. Conclusions

The efficiency of FRET between the CDs and RF was optimized and the main parameters of the FRET process were determined. The excitation wavelength, pH, and donor concentration were optimized and selected. Our results confirm the dependence of the energy transfer efficiency on the acceptor:donor ratio in nanoparticle-based systems. The study of the FRET process at two donor concentrations showed that the energy transfer efficiency decreases as the donor concentration increases. This behavior is due to the configuration of one nanoparticle interacting with several acceptors, which produces an increase in the overlap integral proportional to the number of RF acceptors per CD donor. The results show the importance of modulating the CDs–RF interaction to improve the output signal from FRET. With optimal conditions, the best obtained efficiency of the FRET process was 91%, with a Förster distance (*R_0_*) of 3.3 nm and a distance between the donor and the acceptor (*r*) of 1.8 nm. The results were then applied in the development of an ultrasensitive ratiometric fluorescent sensor for the determination of RF in different beverages. The nanosensor is easy to synthesize and exhibits high sensitivity and selectivity. The nanoprobe is fast, simple to handle, and low-cost. Remarkably, the developed method was applied to the determination of RF in different beverages (an energy drink, green tea, and wine) with recoveries between 96% and 106% and a SDR lower than 4%. The developed nanoprobe method of RF determination can be extended to other samples of importance in the biological, environmental, and food sectors. Moreover, thanks to its outstanding cytocompatibility at the working concentrations, even after long incubation times with cells, the developed platform can also be envisaged for other applications of biological interest, such as intracellular sensing and staining for live cell microscopy.

## Figures and Tables

**Table 1:** FRET parameters.

FRET Parameters					
Conjugate	*J* (cm^3^ L mol^−1^)	*E* (%)	*R*_0_ (nm)	*r* (nm)	*K* (M^−1^)
CDs/RF	3.3 × 10^−15^	91	3.3	1.8	2.7 × 10^4^

**Table 2:** Comparison of different methods for RF determination.

Method/Sensor Material/Detection	Linear Range, µM	Det. Limit, µM	Ref
Cr-doped SnO_2_ nanoparticles electrode/Cyclic Voltammetry	0.2–100	0.107	[8]
Bismuth-film electrode/Cyclic Voltammetry	0.3–0.8	0.1	[13]
Fe_3_O_4_/Graphene oxide electrode/Differential pulse voltammetry	0.3–1.0	0.089	[14]
Capillary Electrophoresis/Laser-Induced Fluorescence	0.001–1.33	0.008	[15]
HPLC/tandem mass spectrometry	0.011–53.1	0.029	[12]
Ag nanoparticles modified with β-CD/Colorimetry	0.16–4.66	0.167	[36]
Spectrophotometry/Absorbance at 440 nm	0.797–123	0.79	[11]
Graphitic carbon nitride/Fluorimetry (FRET)	0.4–10	0.170	[16]
Carbon-dot-codoped N, P/Fluorimetry (FRET)	0.5–50	0.170	[17]
g-CNQDs@Zn-MOF/Fluorimetry (FRET)	0–1	0.015	[18]
Sulfonated graphene/Fluorimetry (FRET)	0.4–10	1.6	[37]
N,S-doped carbon dots/Fluorimetry (FRET)	0.56–7.4	1.9	[19]
Graphene-PEG/Fluorescence Quenching	0–6.3	60	[38]
Molecularly Imprinted Polymer (PIM)/Fluorimetry	Not reported	79.7	[39]
Carbon dots (N, COO^−^)/Fluorimetry (FRET)	0–11	0.025	This work

**Table 3:** Determination of RF in real samples.

Sample	Added (µM)	Found (µM)	Recovered (µM)	Recovered (%)	SD	RSD
Monster Energy	2.5	3.81	6.46	106.30	0.02	3.64
	3.5		7.21	97.26	0.01	1.13
	4.5		8.38	101.60	0.02	2.67
	5.5		9.22	98.37	0.03	3.40
	6.5		1.01	97.59	0.01	0.75
	7.5		1.16	104.50	0.02	1.28
	8.5		1.20	96.58	0.01	0.69
Green tea	2.5	1.33	3.94	104.50	0.02	3.07
	3.5		4.76	97.95	0.01	1.62
	4.5		5.81	99.51	0.01	1.74
	5.5		7.07	104.30	0.01	1.12
	6.5		7.96	101.90	0.02	1.56
	7.5		8.84	101.10	0.04	3.13
	8.5		9.68	98.24	0.02	1.42
White wine	2.5	1.61	4.08	98.80	0.01	1.30
	3.5		5.05	98.32	0.01	1.37
	4.5		6.18	101.60	0.02	1.39
	5.5		7.21	101.80	0.03	1.79
	6.5		8.33	103.50	0.02	1.48
	7.5		8.97	98.23	0.03	1.49
	8.5		1.00	99.06	0.07	3.19

## Data Availability

Not applicable.

## Supplementary Materials

The following are available online at https://www.mdpi.com/article/10.3390/nano11081981/s1, Figure S1: Variation in CDs’ Zeta Potential with pH, Figure S2: Photostability of CDs in water at pH 7.4 under UV light irradiation, Figure S3 (A) UV visible absorption spectrum and (B) fluorescence spectrum of RF (1.0 × 10^−5^ M), Figure S4 (A) Stern–Volmer plot and (B) Stern–Volmer modified plot of the fluorescence quenching and Stern–Volmer analysis and calculation of the binding constant.
