# Supplementary material for: Optimizing the Efficiency of a Cytocompatible Carbon-Dots-Based FRET Platform and Its Application as a Riboflavin Sensor in Beverages
Source: Nanomaterials (Basel). 2021 Jul 31;11(8):1981. doi: 10.3390/nano11081981 (PMC8399497; doi:10.3390/nano11081981)
Supplement: Supplementary file 1 [file nanomaterials-11-01981-s001.zip › nanomaterials-1278190-supplementary.pdf]

nanomaterials

Supplementary Materials

Optimizing the efficiency of a cytocompatible carbon dots-based FRET platform and its application as a riboflavin sensor in beverages

Roberto Sotolongo-García [1], Eustolia Rodríguez-Velázquez [2,3], Manuel Alatorre-Meda [4], Mercedes T. Oropeza-Guzmán [1], Antonio Tirado-Guízar [1] and Georgina Pina-Luis [1*]

[1] Tecnológico Nacional de México/Instituto Tecnológico de Tijuana, Centro de Graduados e Investigación en Química, Blvd. Alberto Limón Padilla S/N, 22500Tijuana, B.C., México; robertosg2989@gmail.com (R.S.-G.); oropeza@tectijuana.mx (M.T.O.-G.); antonio.tirado@tectijuana.edu.mx (A.T.-G.)
[2] Facultad de Odontología, Universidad Autónoma de Baja California, Calzada Universidad 14418, Parque Industrial Internacional, 22390 Tijuana, BC, Mexico; eustolia.rodriguez@uabc.edu.mx (E. R.-V.)
[3] Tecnológico Nacional de México/I. T. Tijuana, Centro de Graduados e Investigación en Química-Grupo de Biomateriales y Nanomedicina, Blvd. Alberto Limón Padilla S/N 22510 Tijuana, B. C., México
[4] Cátedras CONACyT-Tecnológico Nacional de México/I. T. Tijuana, Centro de Graduados e Investigación en Química-Grupo de Biomateriales y Nanomedicina, Blvd. Alberto Limón Padilla S/N, 22510 Tijuana, B. C., México; manuel.alatorre@ tectijuana.edu.mx (M.A.-M.)
* Correspondence: gpinaluis@yahoo.com; gpinaluis@tectijuana.mx (G.P.-L)

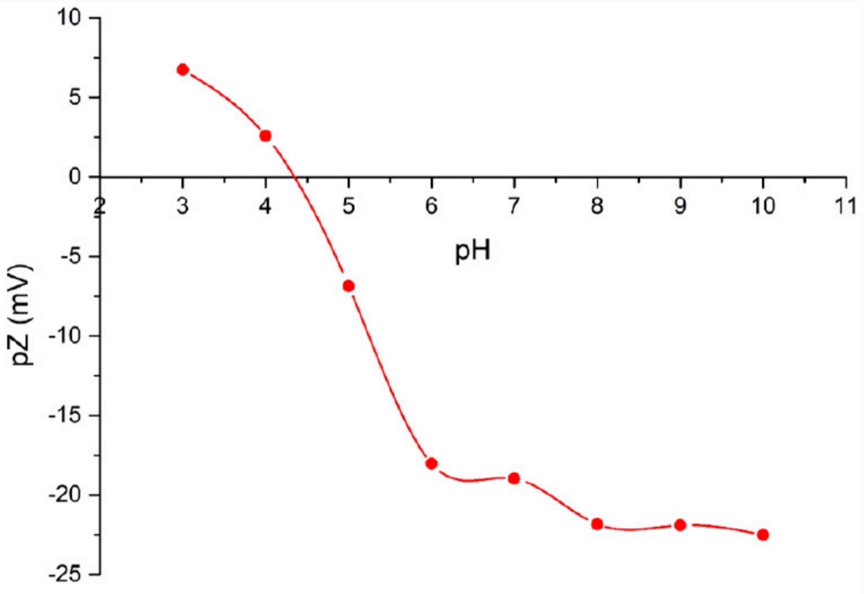

Figure S1. Variation of CDs Zeta Potential with pH.

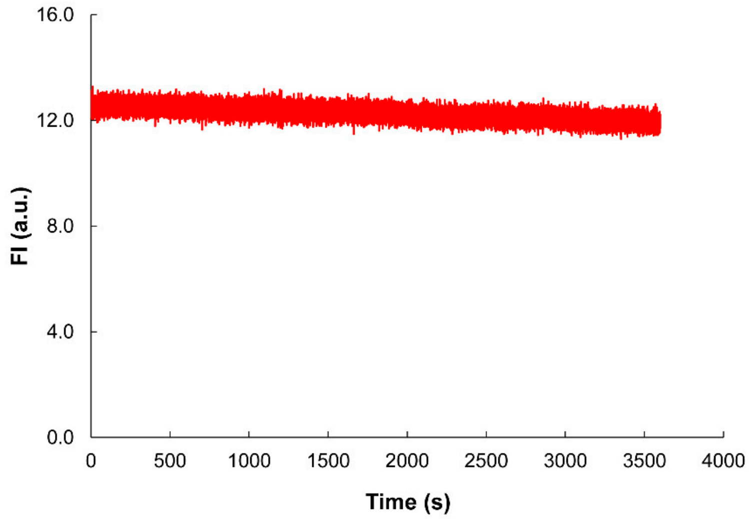

Figure S2. Photostability of CDs in water at pH 7.4 under UV light irradiation.

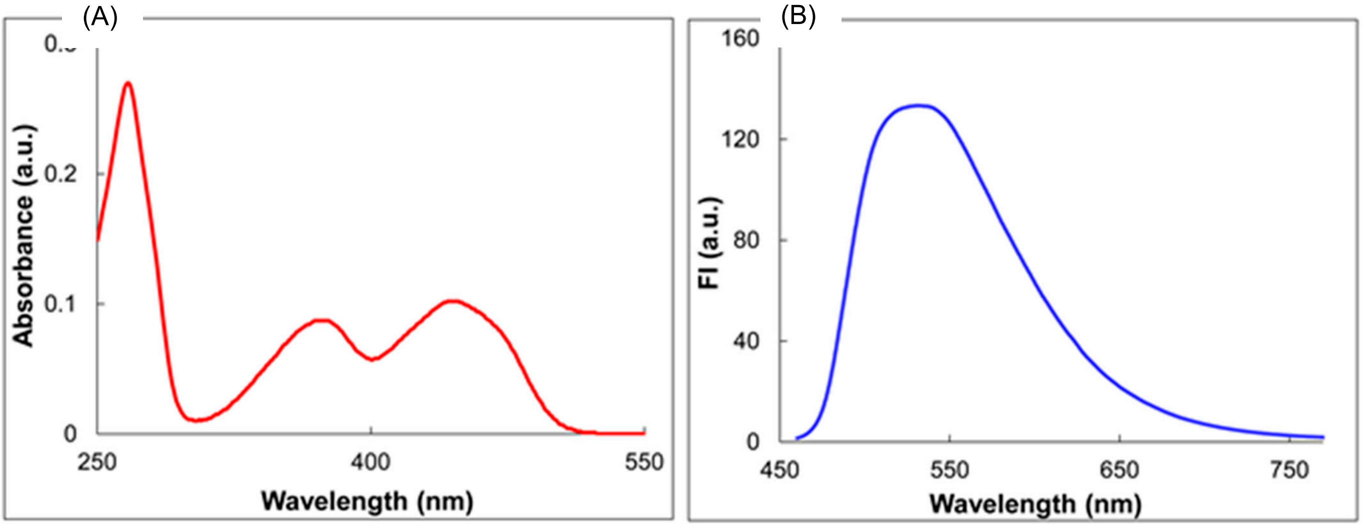

Figure S3. A) UV visible absorption spectrum and B) fluorescence spectrum of RF (1.0x10^{-5} M).

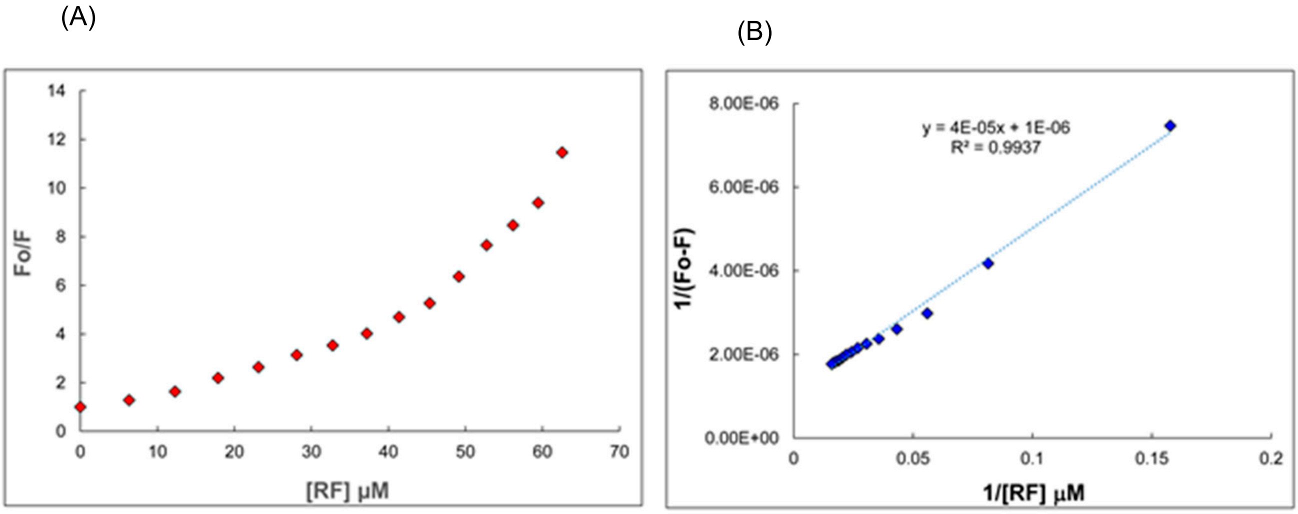

Figure S4. A) Stern Volmer plot and B) Stern Volmer modified plot of the fluorescence quenching.

Stern Volmer analysis and calculation of the binding constant

The Stern Volmer equation (1) was used to describe fluorescence quenching:

$$F_0/F = 1 + K_{SV}[RF] \quad (1)$$

In this equation, F_o and F are the fluorescence intensities observed in the absence and presence of RF (quencher), K_{SV} is the Stern-Volmer constant and $[RF]$ is the concentration of RF [1]. The relative fluorescence intensity, F_o/F displays an upward curvature (Figure S4A), indicating two types of quenching mechanisms (dynamic and static).

To analyze if the interaction between CDs and RF is strong, moderate, or weak, we calculated the modified Stern-Volmer binding constant (K) by Stern-Volmer modified Equation (2) [2]:

$$\frac{1}{F_0 - F} = \frac{1}{F_0} + \frac{1}{KF_0[RF]} \quad (2)$$

The linearity of the Stern Volmer modified equation indicates that there is a contribution of the binding between the fluorophore and quencher to the quenching process. Nonlinearity is indicative that the binding process, is not the dominant mechanism for fluorescence quenching [3]. The calculated value of K is 2.7×10^4 M^{-1} with a correlation coefficient of 0.9937 (Figure S4B). The binding constant greater than 10^4 M^{-1} suggests that there is a moderate interaction of CDs with RF. In our case the K value acquires more relevance since it was obtained in an aqueous competitive medium. Then the value of K shows the high affinity of RF for CD. This is an important result that influences the sensitivity and selectivity of the sensor.

Reference:

[1] Lakowicz, J.R. *Principles of Fluorescence Spectroscopy*; Springer: New York, NY, USA, 2006; pp. 443–472.
[2] Chaves, O. A.; Amorim, A. P. de O.; Larissa H. E. Castro, L. H. E.; Sant' Anna, C. M. R.; de Oliveira, M. C.; Cesarin-Sobrinho, D.; Neto-Ferreira, J. C.; Ferreira, A. B. B. Fluorescence and docking studies of the interaction between human serum albumin and pheophytin. *Molecules* **2015**, *20*, 19526-19539.
[3] Samworth, C. M.; Esposti, M. D.; Lenaz, G. Quenching of the intrinsic tryptophan fluorescence of mitochondrial ubiquinol-cytochrome-c reductase by the binding of ubiquinone. *Eur. J. Biochem*. **1988**, *171*, 81-86.
